# Comparative Sequence–Structural Analysis of TdNV-Korea Entry Factors Identifies Candidate Regions Associated with Host-Range Variation

**DOI:** 10.3390/ijms27146250

**Published:** 2026-07-14

**Authors:** Yoon Ho Park, Rana Kim, Kun-Ho Song, Hyun Suk Jung

**Affiliations:** 1 Department of Biochemistry, College of Natural Sciences, Kangwon National University, Chuncheon 24341, Republic of Korea; yhpark99@kangwon.ac.kr (Y.H.P.); rana1219@kangwon.ac.kr (R.K.); 2 University-Industry Cooperation Foundation, Kangwon National University, Chuncheon 24341, Republic of Korea

**Keywords:** *Oryctes rhinoceros* nudivirus, per os infectivity factor, host adaptation, cross-species transmission, intrinsically disordered region, electrostatic surface potential

## Abstract

Emerging viral variants can affect both commercial insect production and wild insect populations, highlighting the need to identify molecular features associated with viral host range. TdNV-Korea, isolated from the Korean rhinoceros beetle *Allomyrina dichotoma* (syn. *Trypoxylus dichotomus*), represents a Korean isolate within the *Oryctes rhinoceros* nudivirus (OrNV) lineage associated with infection of a non-*Oryctes* scarab host. To define candidate features associated with host-range variation, we compared the sequence and predicted structural properties of viral entry factors across OrNV-related isolates using sequence analysis, structural modeling, and electrostatic surface analysis. The major capsid protein VP39 was highly conserved and served as a comparative baseline, whereas the per os infectivity factors GP106 and GP126 showed greater divergence despite their expected constraints during oral entry. GP106 retained an invariant chitin-binding domain, with substitutions restricted to a peripheral C-terminal region, whereas GP126 concentrated most substitutions within a predicted disordered interdomain segment associated with a localized electrostatic shift on a candidate host-interaction surface. Because functional assays were not performed, these findings should be interpreted as a hypothesis-generating model in which two entry factors follow contrasting modes of sequence change. GP126, and secondarily, the GP106 C-terminal region are prioritized for future experimental validation of nudivirus host-range determinants.

## 1. Introduction

Insect agriculture has emerged as an important sector for the sustainable production of alternative proteins and biomaterials, but its expansion is accompanied by pathogen-related biosecurity risks [1]. These threats include nudiviruses, members of the family Nudiviridae, which are enveloped, rod-shaped double-stranded DNA viruses that infect diverse insect orders, including Coleoptera, Lepidoptera, Diptera, and Orthoptera [1]. The *Oryctes rhinoceros* nudivirus (OrNV) has been extensively used as a biological control agent against its natural host, the coconut rhinoceros beetle (*O. rhinoceros*), and has historically been associated with beetles of the genus *Oryctes* [2,3]. However, the biological properties that support OrNV transmission and oral infection may also create opportunities for infection of non-target scarab beetles. Such a host transition was reported in 2015, when OrNV was associated with severe disease in the Korean rhinoceros beetle, *Allomyrina dichotoma* (syn. *Trypoxylus dichotomus*), a scarab beetle outside the genus *Oryctes* [4].

Genomic characterization of the corresponding isolate, Trypoxylus dichotomus nudivirus Korea (TdNV-Korea; GenBank BK063656), showed that its overall genome structure is highly conserved relative to other OrNV isolates. Nevertheless, amino-acid substitutions are distributed across many nudivirus core genes [5]. This pattern of broad conservation interspersed with localized divergence raises the question of which viral proteins are most likely to contribute to infection of a host from a different genus and by what mechanisms. Because infection is initiated after ingestion and requires passage across the midgut barrier, proteins acting at the oral-entry step are plausible candidates. In nudiviruses and the closely related baculoviruses, this step is thought to be mediated by a conserved set of envelope-associated per os infectivity factors (PIFs), which are essential for establishing infection in the insect midgut [6].

PIFs assemble into a multiprotein complex on the surface of occlusion-derived virions. Within this complex, PIF1–PIF3 form a stable core, while components such as P74/PIF0 associate with the core and contribute to virion binding to midgut epithelial cells [7,8,9]. Within the OrNV genome, two factors are of particular interest because they are positioned at the virus–host entry interface: GP106 and GP126. GP106 carries a chitin-binding domain that may mediate interaction with the peritrophic matrix, a chitin-rich physical barrier in the insect midgut [10], whereas GP126 is homologous to baculovirus P74/PIF0 and is proposed to participate in initial receptor binding on midgut epithelial cells [6,11,12]. Genomic studies have identified amino-acid substitutions across many TdNV-Korea genes, including these entry factors, and comparative analyses of OrNV isolates have suggested that such variation may be associated with differences in pathogenicity or transmissibility [5,13]. These reports, however, remain primarily sequence-based, and the potential molecular features associated with TdNV-Korea infection of a divergent host have not been examined. In particular, it remains unknown whether substitutions in GP106 and GP126 occur within functionally important regions, information that would be needed to connect sequence variation to possible host adaptation.

To address this gap, we examined how substitutions in GP106 and GP126 are positioned within the three-dimensional architecture of each protein and whether they alter functionally relevant surfaces. Recent advances in protein structure prediction, particularly AlphaFold, make such analyses feasible, and the per-residue confidence metric (pLDDT) can help identify regions of low structural confidence that often overlap with intrinsically disordered regions (IDRs) [14,15,16,17]. We therefore integrated multi-isolate sequence alignments, maximum-likelihood phylogenetics, Foldseek-based domain annotation, AlphaFold3 structural modeling supported by homology searches, and APBS electrostatic surface potential maps to characterize candidate sequence–structural differences in these entry factors. Using this integrative approach, our analyses suggest that GP106 and GP126 exhibit contrasting modes of sequence divergence and that localized charge-altering substitutions in the GP126 disordered region define a candidate region for future functional testing. Together, these analyses provide a focused, hypothesis-generating framework for prioritizing entry-factor regions that may be relevant to host-associated variation in nudiviruses.

## 2. Results

### 2.1. Localized Divergence of Entry Factors Against a Conserved Capsid Baseline

**Figure 1:**
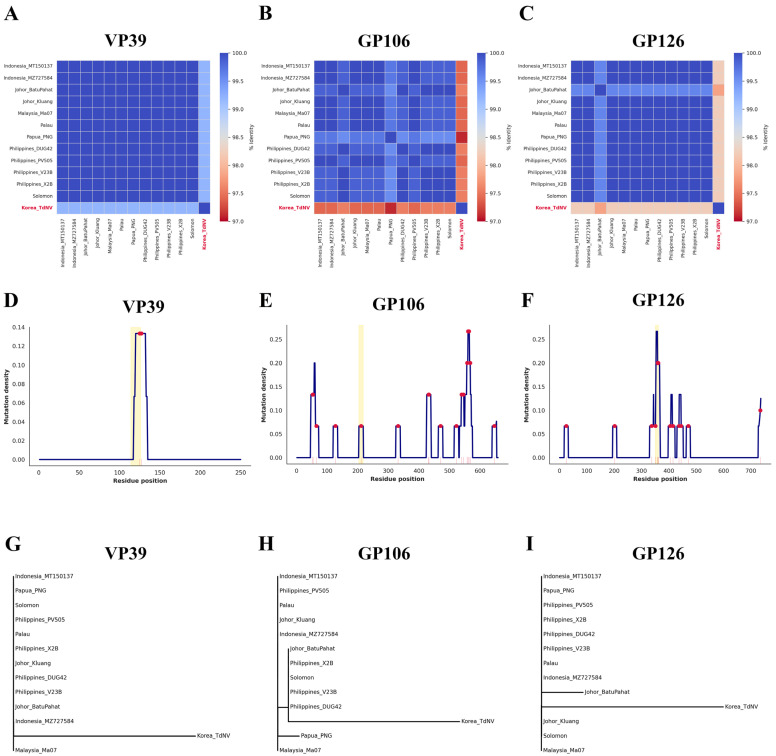
Sequence divergence of VP39, GP106, and GP126 across 13 OrNV isolates. (**A**–**C**) Pairwise amino-acid identity matrices for VP39 (**A**), GP106 (**B**), and GP126 (**C**); color scale indicates percent identity (97.0–100%). TdNV-Korea is highlighted in red. (**D**–**F**) Per-residue mutation density along VP39 (**D**), GP106 (**E**), and GP126 (**F**), computed for the TdNV-Korea sequence relative to the reference consensus using a 15-aa window; red points mark local maxima. In panels E and F, the yellow highlighted region indicates the emphasized sequence segment, and the thin red marker denotes the position of the mutated residue in the TdNV-Korea sequence. (**G**–**I**) Maximum-likelihood phylogenetic trees of VP39 (**G**), GP106 (**H**), and GP126 (**I**), inferred under the best-fit substitution model and rooted on Malaysia Ma07. Branch lengths represent substitutions per site.

To place entry-factor variation against a conserved structural baseline, we compared the major capsid protein VP39 with the entry factors GP106 and GP126 across 13 geographical isolates. VP39 was highly conserved, with TdNV-Korea sharing 99.2% identity with the Asian cluster and forming a very short phylogenetic branch, consistent with strong conservation of the structural core (Figure 1A,D,G) [18]. In contrast, GP106 and GP126 showed lower identities, 97.09% and 97.82%, respectively, with 17 and 13 substitutions and longer TdNV-Korea branches (Figure 1B,C,H,I). The per-residue mutation-density plot further showed that these substitutions were unevenly distributed and concentrated in discrete density peaks (Figure 1E,F), indicating localized divergence in the entry factors.

### 2.2. Mutation Hotspots Map to Distinct Domain Locations in GP106 and GP126

**Figure 2:**
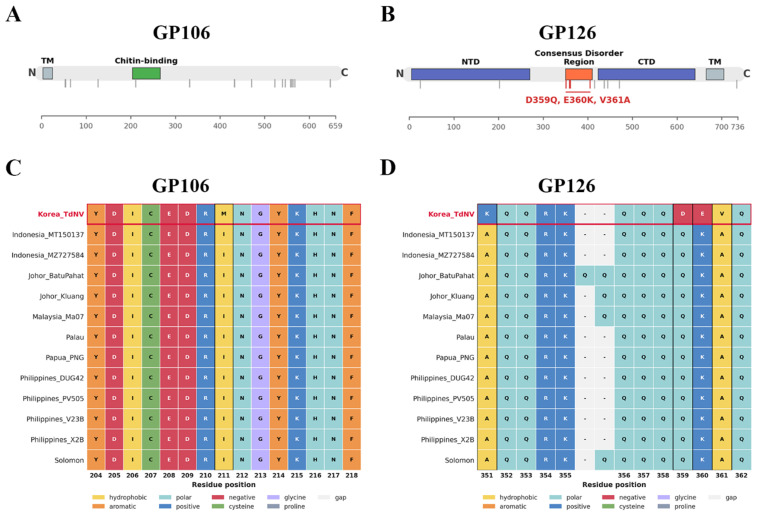
Domain mapping and alignment of representative regions in GP106 and GP126. (**A**,**B**) Linear domain organization of GP106 ((**A**); 659 aa) and GP126 ((**B**); 736 aa) from InterProScan. GP106 features include an N-terminal transmembrane segment (TM) and a chitin-binding type-2 domain (residues 204–266). GP126 features include the Baculo_p74_N domain (Pfam PF08404; residues 5–270), the Baculo_p74 conserved region (PF04583; residues 423–640), transmembrane segments (TM), and the MobiDB-lite consensus disorder region (residues 350–410). Vertical ticks below each schematic mark the positions of TdNV-Korea amino-acid substitutions; the GP126 disorder-region substitutions D359Q, E360K, and V361A are labeled. (**C**) Multiple sequence alignment of the GP106 chitin-binding domain region (residues 204–218) across 13 isolates. (**D**) Multiple sequence alignment of the GP126 disorder region (residues 351–362). TdNV-Korea is boxed in red. Residues are colored by physicochemical class (hydrophobic, aromatic, polar, positive, negative, glycine, proline, cysteine, gap) and letters indicate the amino-acid identities at each position.

To determine where entry-factor substitutions occur relative to functional domains, we mapped each substitution onto the domain architecture of GP106 and GP126 (Figure 2). InterProScan (web server, EMBL-EBI; accessed in 17 May 2026) analysis identified an N-terminal transmembrane segment in GP106 (residues 3–25) and a chitin-binding type-2 domain (PROSITE PS50940; residues 204–266). TdNV-Korea substitutions were distributed along GP106 but were absent from the chitin-binding domain, which was invariant across all 13 isolates. The highest-density mutation window instead mapped to the C-terminal region (residues 555–567), outside any annotated domain. For GP126 (736 aa), InterProScan identified an N-terminal Baculo_p74_N domain (Pfam PF08404; residues 5–270) and a C-terminal Baculo_p74 conserved region (PF04583; residues 423–640). Predicted transmembrane helices followed the C-terminal conserved region, and MobiDB-lite annotated the intervening residues 350–410 as a consensus disorder region (Figure 2B). Of the 13 TdNV-Korea substitutions in GP126, 11 fell within this 350–410 disorder region (Figure 2D), including D359Q, E360K, and V361A, which alter the local physicochemical profile of residues conserved in the reference isolates. Thus, the two entry factors differed in the localization of their substitutions: GP106 showed no substitutions in its chitin-binding domain, whereas GP126 concentrated substitutions within a predicted disorder region between its conserved P74 domains.

### 2.3. Structural Modeling Localizes GP126 Substitutions to a Predicted Disordered Interdomain Region

**Figure 3:**
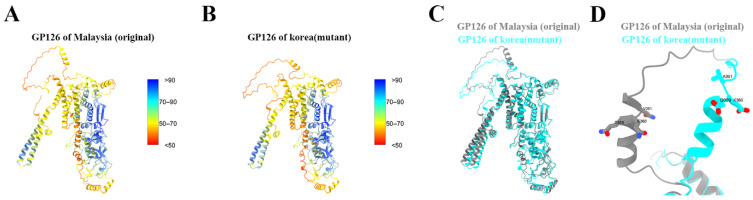
AlphaFold3 structural models and superimposition of GP126 from Malaysia Ma07 and TdNV-Korea. (**A**,**B**) AlphaFold3-predicted models of GP126 from Malaysia Ma07 (**A**) and TdNV-Korea (**B**), colored by per-residue confidence (pLDDT; red <50, orange 50–70, yellow–green 70–90, blue >90). (**C**) Structural superimposition of the Malaysia (gray) and TdNV-Korea (cyan) models. (**D**) Close-up of the 340–410 aa region from the superimposition in (**C**), showing the substituted residues (Malaysia: D359, E360, V361; TdNV-Korea: Q359, K360, A361) as sticks.

To examine the structural context of the GP126 substitutions, we generated AlphaFold3 models of the Malaysia Ma07 and TdNV-Korea isoforms and compared their confidence profiles and predicted folds (Figure 3). Foldseek searches against the BFVD identified GP126 as a member of the baculovirus P74 (PIF0) superfamily, returning full-length, high-confidence matches to P74-type proteins (probability = 1.0), consistent with the Pfam assignment of its Baculo_p74_N and Baculo_p74 domains. In both AlphaFold3 models, the N- and C-terminal domains were predicted with high confidence (pLDDT > 90), whereas the 350–410 aa segment showed lower confidence, with pLDDT values of approximately 50–70 (Figure 3A,B). This low-confidence segment coincides with the MobiDB-lite consensus disorder region, supporting its interpretation as a likely intrinsically disordered region, although local coordinates should be treated with caution [16,17]. Structural superimposition showed that the N- and C-terminal domains aligned with minimal deviation, indicating a conserved overall fold between isolates (Figure 3C). By contrast, the low-confidence 350–410 aa segment differed between models, with the substituted residues Q359, K360, and A361 in TdNV-Korea corresponding to D359, E360, and V361 in Malaysia (Figure 3D). Given the low pLDDT, the coordinates of this segment were interpreted qualitatively.

### 2.4. Predicted Local Electrostatic Shifts in the GP126 340–410 aa Region

**Figure 4:**
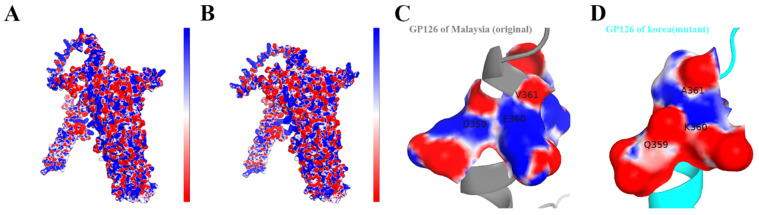
Electrostatic surface potential of GP126 from Malaysia Ma07 and TdNV-Korea. (**A**,**B**) APBS electrostatic surface potential mapped onto the full GP126 models of Malaysia Ma07 (**A**) and TdNV-Korea (**B**); surfaces are colored from negative (red) to positive (blue) potential over a range of −5 to +5 kT/e. (**C**,**D**) Close-up views of the GP126 340–410 aa region in Malaysia Ma07 (**C**) and TdNV-Korea (**D**), with residues 359–361 labeled (Malaysia: D359, E360, V361; TdNV-Korea: Q359, K360, A361). The same color scale (−5 to +5 kT/e) is applied in all panels.

To assess whether the GP126 substitutions may alter predicted local surface properties, we computed APBS electrostatic surface potentials for the Malaysia Ma07 and TdNV-Korea models (Figure 4) [19]. At the whole-protein level, the overall charge distribution was broadly similar between isolates, consistent with the conserved core fold (Figure 4A,B). In the 340–410 aa region, however, the local electrostatic pattern differed between the two isoforms (Figure 4C,D). In Malaysia Ma07, residues D359 and E360 were associated with a negatively charged surface patch, whereas the corresponding D359Q and E360K substitutions in TdNV-Korea reduced or reversed this local negative character. Together with V361A, these substitutions were predicted to shift the local surface potential around residues 359–361 toward a more neutral or positive profile. These results suggest a localized electrostatic change in the GP126 340–410 aa region, while the broader surface architecture remains comparable between isolates.

## 3. Discussion

This study compared the sequence–structural features of three TdNV-Korea proteins and found that the two envelope-associated entry factors, GP106 and GP126, exhibit contrasting patterns of divergence relative to the conserved capsid protein VP39. GP106 showed an invariant chitin-binding domain (residues 204–266) and substitutions restricted to a peripheral C-terminal region, whereas GP126 concentrated 11 of its 13 substitutions within a 350–410 aa segment annotated as a consensus disorder region between two conserved P74 domains. These patterns are consistent with the distinct functional roles proposed for the two proteins. Because the peritrophic matrix targeted by the GP106 chitin-binding domain is a broadly conserved chitin-rich barrier [10], this domain may experience stronger functional constraint across related hosts. In contrast, GP126 is homologous to baculovirus P74/PIF0, an entry factor implicated in ODV binding to host midgut epithelial cells and in early per os infection [6,9]. Infection of *A. dichotoma*, a non-*Oryctes* host, could therefore be associated with stronger host-specific constraints on GP126 than on the more structurally conserved capsid protein, consistent with the localization pattern observed here. Notably, baculovirus P74 provides functional precedent that the interdomain region of a P74-type entry factor can contribute to oral infection. P74 undergoes proteolytic cleavage during ODV release and in the insect midgut, and the resulting fragments remain associated with the PIF complex, suggesting that cleavage may activate P74 or expose functional regions required for per os infectivity [11]. More recently, AcMNPV P74 was shown to be cleaved at R325/R334, located between the N- and C-conserved P74 domains, by proteinases from both occlusion bodies and host brush border membrane vesicles; mutation of these sites significantly reduced oral infectivity, and cleavage was proposed to expose a potential fusion peptide for oral infection [12]. Although the GP126 350–410 aa segment has not been experimentally tested for cleavage, receptor binding, or fusion, its position between conserved P74-like domains, predicted disorder, concentration of TdNV-Korea substitutions, and localized electrostatic shift support its prioritization as a candidate region for future functional analysis.

Several independent observations support the interpretation of the GP126 350–410 aa region as a candidate intrinsically disordered region. The region was annotated as a consensus disorder region by MobiDB-lite, lies between two structured Pfam domains, and showed consistently low AlphaFold3 pLDDT scores [16,17]. Foldseek independently identified GP126 as a P74/PIF0 superfamily protein, supporting the conservation of the flanking scaffold while this segment lacks a stable predicted fold. The enrichment of substitutions within a disordered, surface-exposed region is consistent with broader observations that viral intrinsically disordered regions can accommodate sequence change with limited destabilization of the overall structure [18,20,21]. The local electrostatic shift predicted in this region suggests a testable hypothesis: D359Q, E360K, and V361A reduce or reverse the negative surface character present in the Malaysia reference, potentially altering electrostatic complementarity with a host midgut receptor [19]. We emphasize that this remains a hypothesis-generating observation, as the present data establish a charge change at a predicted surface rather than a demonstrated change in binding, oral infectivity, or midgut attachment.

The domain organization of GP126 parallels that of baculovirus P74/PIF0, which contains conserved P74 domains and a C-terminal membrane-anchoring region and associates with the PIF complex built around the PIF1-PIF3 core [7,8]. Together with baculovirus studies showing that P74 is required for ODV binding and undergoes entry-relevant proteolytic activation, this architecture supports a model in which a conserved P74-like scaffold presents a variable interdomain segment for host-associated entry interactions [6,9,11,12]. In GP126, the Baculo_p74_N domain (residues 5–270) provides the N-terminal scaffold, and the Baculo_p74 region (residues 423–640) with its transmembrane helices forms the C-terminal anchor, while the intervening disorder region (residues 350–410) offers a variable surface. This conserved-scaffold-plus-variable-surface arrangement may represent a recurring entry-factor architecture that preserves core function while permitting surface variation. These observations may also have practical relevance for OrNV biocontrol, where the emergence of the CRB-G haplotype with reduced susceptibility to classical isolates has renewed interest in matching viral isolates to target beetle populations [3,22,23]. If GP126 surface properties influence host compatibility, sequence variation and predicted electrostatic features of the 350–410 region may be useful candidate parameters for isolate selection and surveillance of newly detected variants in reared scarabaeids such as *A. dichotoma*.

Several limitations should be noted. The structures analyzed here are AlphaFold3 predictions; although the well-folded P74 domains were modeled with high confidence, the 350–410 aa region is low-confidence and should be treated qualitatively as one of many possible conformational states [15,16,24]. The APBS v3.4.1 calculations are based on static models and do not capture conformational dynamics, the membrane environment, or post-translational modifications; therefore, they indicate charge distribution rather than binding energetics. We have not experimentally tested whether the GP126 substitutions alter receptor binding, oral infectivity, or interaction with the midgut barrier. Future work should therefore test these predictions directly using site-directed substitutions in the GP126 350–410 region and the GP106 C-terminal hotspot, followed by oral infection assays in relevant beetle hosts. Midgut binding and peritrophic matrix interaction assays would also be needed to determine whether the predicted electrostatic differences alter viral attachment or passage through the midgut environment. Together, the present analyses should be viewed as a focused hypothesis-generating study that integrates comparative sequence analysis, domain annotation, structural modeling, and electrostatic surface analysis to prioritize candidate entry-factor regions for experimental validation, rather than as direct evidence that these substitutions determine host specificity.

## 4. Materials and Methods

### 4.1. Viral Sequence Retrieval and Dataset Curation

The complete amino-acid sequences of three viral proteins, the core capsid protein VP39 and the per os infectivity factors GP106 and GP126, were retrieved from the NCBI GenBank database. The dataset comprised the newly isolated TdNV-Korea (accession no. BK063656) and 12 geographically distinct reference isolates: the type isolates Malaysia Ma07 (EU747721), Indonesia (MT150137, MZ727584), Solomon Islands (MN623374), Palau (MW298154), Philippines (OP831188, OP831190, OP831191, OP831186), Johor (ON931347, ON931348), and Papua New Guinea (OP831187). The Tanzania isolate (OP831189) was excluded during curation to focus the analysis on divergence within the Asian cluster. Multiple sequence alignments were generated independently for each protein using MAFFT v7 with the L-INS-i iterative refinement strategy [25].

### 4.2. Maximum-Likelihood Phylogenetic Reconstruction

Phylogenetic relationships were reconstructed using maximum likelihood in IQ-TREE v2.0.7 [26]. The best-fit amino acid substitution model for each alignment was selected with ModelFinder based on the Bayesian information criterion [27], yielding the FLU model for GP106. Branch support was assessed using the ultrafast bootstrap approximation (UFBoot2) with 1000 replicates [28]. Trees were rooted on the reference isolate Malaysia Ma07, and topologies and support values were visualized with the Biopython Phylo module (version 1.87) [29].

### 4.3. Sequence Identity and Per-Residue Mutation Density

Pairwise amino acid identities among the 13 curated isolates were calculated from the MAFFT alignments and visualized as heatmaps using Seaborn 0.13.2 [30]. To locate substitution clusters, per-residue mutation density was computed along the TdNV-Korea sequence relative to the reference-cluster consensus using a 15-amino-acid sliding window. The highest-density windows, residues 555–567 in GP106 and residues 351–362 in GP126, were defined as mutation hotspots. Substitutions within these windows were annotated and classified by side-chain physicochemical class (hydrophobic, polar, aromatic, acidic, and basic).

### 4.4. Domain Architecture and Disorder Annotation

Domain architecture was annotated with InterProScan v5 [31]. Structured domains were assigned from Pfam signatures (Baculo_p74_N, PF08404; Baculo_p74, PF04583) and the PROSITE chitin-binding type-2 profile (PS50940), and transmembrane segments were predicted by TMHMM Server v 2.0 (web application, DTU Health Tech; accessed in 17 May 2026) and Phobius 1.01 (web server, Stockholm University; accessed in 17 May 2026). Intrinsically disordered regions were identified using MobiDB-lite 4.0 (web server; accessed in 17 May 2026) consensus disorder prediction [32], which assigned residues 350–410 of GP126 as a consensus disorder region.

### 4.5. Structural Similarity Search

Structural homology was assessed with Foldseek v.10 (10-941cd33) [33] by querying the AlphaFold3 v3.0.3 models against the Big Fantastic Virus Database (BFVD) [34]. GP126 returned full-length, high-confidence matches (probability = 1.0) to baculovirus P74 (PIF0) proteins, and GP106 matched chitin-binding and p95/ORF75-type proteins, supporting the InterProScan domain assignments.

### 4.6. Structure Prediction and Electrostatic Surface Analysis

Three-dimensional models of GP126 from Malaysia Ma07 and TdNV-Korea were predicted using the AlphaFold3 server [15], and per-residue confidence (pLDDT) was used as an indicator of local structural reliability and potential disorder propensity [16,17]. The resulting models were superimposed in PyMOL (version 2.4.1) [35] to compare the overall fold and the conformation of the 350–410 region. Electrostatic surface potentials were calculated using the APBS plugin in PyMOL [19] and visualized over a uniform color scale of −5 to +5 kT/e, applied identically to all models to enable direct comparison.

## Data Availability

The primary sequence data used in this study are publicly available in the NCBI GenBank database under accession numbers BK063656, EU747721, MT150137, MZ727584, MN623374, MW298154, OP831188, OP831190, OP831191, OP831186, ON931347, ON931348, and OP831187. The processed and derived data generated in this article, including sequence alignments, mutation-density analyses, phylogenetic trees, domain and disorder annotations, structural models, electrostatic surface analyses, and figure source data, are available from the corresponding author upon reasonable request. [NCBI GenBank] [https://www.ncbi.nlm.nih.gov/genbank/] (accessed on 17 May 2026) [BK063656; EU747721; MT150137; MZ727584; MN623374; MW298154; MW298154; OP831188; OP831190; OP831191; OP831186; ON931347; ON931348; OP831].
